# Elevated baseline work rate slows pulmonary oxygen uptake kinetics and decreases critical power during upright cycle exercise

**DOI:** 10.14814/phy2.13802

**Published:** 2018-07-23

**Authors:** Richie P. Goulding, Denise M. Roche, Simon Marwood

**Affiliations:** ^1^ School of Health Sciences Liverpool Hope University Liverpool United Kingdom

**Keywords:** Critical power, exercise tolerance, near infrared spectroscopy, oxygen uptake kinetics, work‐to‐work exercise

## Abstract

Critical power is a fundamental parameter defining high‐intensity exercise tolerance, and is related to the phase II time constant of pulmonary oxygen uptake kinetics ($\tau_{\dot{V}O_{2}}$). Whether this relationship is causative is presently unclear. This study determined the impact of raised baseline work rate, which increases $\tau_{\dot{V}O_{2}}$, on critical power during upright cycle exercise. Critical power was determined via four constant‐power exercise tests to exhaustion in two conditions: (1) with exercise initiated from an unloaded cycling baseline (U→S), and (2) with exercise initiated from a baseline work rate of 90% of the gas exchange threshold (M→S). During these exercise transitions, $\tau_{\dot{V}O_{2}}$ and the time constant of muscle deoxyhemoglobin kinetics (*τ*_[HHb + Mb]_) (the latter via near‐infrared spectroscopy) were determined. In M→S, critical power was lower (M→S = 203 ± 44 W vs. U→S = 213 ± 45 W, *P *=* *0.011) and $\tau_{\dot{V}O_{2}}$ was greater (M→S = 51 ± 14 sec vs. U→S = 34 ± 16 sec, *P *=* *0.002) when compared with U→S. Additionally, *τ*_[HHb + Mb]_ was greater in M→S compared with U→S (M→S = 28 ± 7 sec vs. U→S = 14 ± 7 sec, *P *=* *0.007). The increase in $\tau_{\dot{V}O_{2}}$ and concomitant reduction in critical power in M→S compared with U→S suggests a causal relationship between these two parameters. However, that *τ*_[HHb + Mb]_ was greater in M→S exculpates reduced oxygen availability as being a confounding factor. These data therefore provide the first experimental evidence that $\tau_{\dot{V}O_{2}}$ is an independent determinant of critical power. *Keywords* critical power, exercise tolerance, oxygen uptake kinetics, power‐duration relationship, muscle deoxyhemoglobin kinetics, work‐to‐work exercise.

## Introduction

The hyperbolic relationship between external power and the tolerable duration of exercise (power‐duration relationship) accurately characterizes exercise tolerance for durations ranging from ~2 to 30 min in humans, and is highly conserved across species and modes of exercise (Monod and Scherrer 1965; Moritani et al. 1981; Full and Herreid 1983; Full 1986; Billat et al. 2005; Jones et al. 2008; Burnley 2009; Copp et al. 2010). The power asymptote of this relationship has been termed critical power, and the curvature constant, representing the finite quantity of work available above critical power, has been termed W' (Monod and Scherrer 1965; Moritani et al. 1981; Poole et al. 1988). Critical power represents the highest work rate at which a steady state is attainable for pulmonary $\dot{V}O_{2}$, arterial blood acid‐base status [hydrogen ions (H^+^), lactate (L^−^), and bicarbonate], and intramuscular phosphates [phosphocreatine [PCr] and inorganic phosphate (*P*_i_)] (Poole et al. 1988; Jones et al. 2008; Vanhatalo et al. 2016). Hence, critical power is the highest work rate sustainable without progressive contributions from anaerobic metabolism (Coats et al. 2003; Barker et al. 2006; Chidnok et al. 2013) and is thus an important parameter in defining tolerance to high‐intensity exercise. However, the physiological determinants of critical power have yet to be explicated.

Upon transition from rest to a square‐wave bout of cycle exercise, following a short delay reflecting muscle‐to‐lung venous blood transit time (Whipp et al. 1982), $\dot{V}O_{2}$ rises in an exponential‐like fashion to match the ATP demands of the contracting myocytes. Murgatroyd et al. (2011) previously showed that the time constant of the fundamental phase of oxygen uptake kinetics ($\tau_{\dot{V}O_{2}}$) was strongly related to critical power. However, it was not clear whether this relationship was causal or whether the association arose from both parameters sharing common physiological determinants related to, for instance, training status. Recent work from our laboratory has suggested that $\tau_{\dot{V}O_{2}}$ is an independent determinant of critical power (Goulding et al. 2017). We demonstrated that during supine exercise, a prior bout of high‐intensity “priming” exercise reduced $\tau_{\dot{V}O_{2}}$ (i.e., the $\dot{V}O_{2}$ kinetics were faster) and increased critical power, suggesting that $\tau_{\dot{V}O_{2}}$ is an independent determinant of critical power. However, the concomitant reduction in $\tau_{\dot{V}O_{2}}$ and increase in critical power in the supine position (Goulding et al. 2017) could alternatively have been due to these parameters sharing physiological determinants that were both upregulated as a result of priming exercise. Indeed, muscle perfusion pressure and O_2_ delivery are normally impaired during supine exercise (Karlsson et al. 1975; Convertino et al. 1984; Koga et al. 1999; Jones et al. 2006). Therefore, the increase in critical power following priming exercise in this body position may have been due to the improved O_2_ delivery that attended priming exercise during the transition from rest to exercise, independent of the associated faster $\dot{V}O_{2}$ kinetics. To determine the independence of $\tau_{\dot{V}O_{2}}$ as a determinant of critical power, whether in the upright or supine position, it is therefore necessary to manipulate $\tau_{\dot{V}O_{2}}$ independently of alterations to O_2_ availability.

During exercise transitions initiated from an elevated metabolic rate in the upright position (i.e., “work‐to‐work” exercise), $\tau_{\dot{V}O_{2}}$ has been reported to be increased compared to transitions from a baseline of unloaded cycling (Hughson and Morrissey 1982; di Prampero et al. 1989; Brittain et al. 2001; Wilkerson and Jones 2006, 2007; DiMenna et al. 2010a; Dimenna et al. 2010b; Bowen et al. 2011; Breese et al. 2013). Possible causes of these slowed $\dot{V}O_{2}$ kinetics with work‐to‐work exercise are: (1) the recruitment of a greater proportion of higher‐order motor units which possess slower $\dot{V}O_{2}$ kinetics (Brittain et al. 2001); or (2) the raised work rate resulting in an altered cellular energetic state in already activated fibers (i.e., reduced *P*O_2_ and [PCr], increased [ADP] and [P_i_], less negative changes in the Gibbs free energy of ATP hydrolysis; Δ*G*_ATP_); (Meyer and Foley 1996) slowing the rate of oxidative phosphorylation during the subsequent increase in work rate (Barstow et al. 1994; Glancy et al. 2008; Kemp 2008). Importantly, muscle oxygenation is enhanced prior to the onset of exercise during work‐to‐work transitions (Bowen et al. 2011; Spencer et al. 2011), and pump‐perfusion of canine hindlimb muscle to rates of O_2_ delivery that exceed the steady‐state O_2_ requirements does not reverse the work‐to‐work exercise effect (Wüst et al. 2014). Hence, the lengthening of $\tau_{\dot{V}O_{2}}$ observed with work‐to‐work exercise is not considered to be related to a slower adjustment of O_2_ delivery (DiMenna et al. 2008; Bowen et al. 2011; Spencer et al. 2011; Wüst et al. 2014). The work‐to‐work exercise model therefore represents an ideal means with which to investigate the dependence of critical power on $\tau_{\dot{V}O_{2}}$, independent of changes in O_2_ delivery.

The aim of this study was therefore to assess the impact of raised baseline work rate on pulmonary $\dot{V}O_{2}$ kinetics and critical power during upright cycle exercise. We hypothesized that severe‐intensity exercise transitions from an elevated baseline work rate would result in (1) a greater $\tau_{\dot{V}O_{2}}$ compared with transitions from an unloaded baseline, and (2) a lower critical power. In order to provide insight into the physiological mechanisms underpinning any differences observed, heart rate kinetics were determined and muscle deoxygenation status was also measured via near‐infrared spectroscopy.

## Materials and Methods

### Participants

Seven healthy male participants (mean ± SD, age = 25 ± 3 years; height = 178 ± 6 cm; mass = 77 ± 7 kg) volunteered, providing written informed consent to participate. The study was approved by the Liverpool Hope University Research Ethics Committee in accordance with the Declaration of Helsinki. All participants were recreationally physically active, but not highly trained. All benefits and risks of the protocol, as well as rights to confidentiality and withdrawal, were explained to each participant prior to participation. Participants were asked not to consume alcohol or caffeine within the preceding 24 and 3 h, respectively, prior to each test, as well as to not perform strenuous exercise within the preceding 24 h and to arrive 3 h postprandial.

### Procedures

All tests took place in a well‐ventilated laboratory that was maintained between 18 and 21°C. Participants visited the laboratory on nine occasions during a 3–6 week period, with each test scheduled at the same time of day (±2 h) and with at least 24 h between visits. Participants completed one preliminary trial and eight experimental trials. All exercise tests were performed on an electronically braked cycle ergometer (Lode Excalibur Sport, Groningen, The Netherlands). Saddle and handlebar height/ angle were recorded at the first visit and replicated during each subsequent visit for each individual participant. Throughout all exercise tests, participants were instructed to maintain a cadence of 80 rev min^−1^, and exhaustion was defined as when the participant's cadence dropped below 70 rev min^−1^. Time to exhaustion was measured to the nearest second in all tests.

### Preliminary trial

Upon arrival to the laboratory, participant's height and weight were recorded. Following this, each participant undertook an incremental ramp test until the limit of tolerance to establish $\dot{V}O_{2}\text{max}$, the gas exchange threshold (GET), and the power outputs for subsequent tests. The ramp test consisted of 3‐min of baseline pedaling at 30 W, followed by a continuous, ramped increase in power output of 30 W min^−1^ until the limit of tolerance was established. Gas exchange and ventilatory variables were measured continuously at the mouth breath‐by‐breath throughout each test. $\dot{V}O_{2}\text{max}$was defined as the highest $\dot{V}O_{2}$ value measured over 30 sec. The GET was taken as a non‐invasive estimate of the lactate threshold using a collection of previously established criteria (Beaver et al. 1986); including (1) a disproportionate rise in CO_2_ production ($\dot{V}{\text{CO}}_{2}$) relative to $\dot{V}O_{2}$; (2) an increase in minute ventilation ($\dot{V}E$) relative to $\dot{V}O_{2}$ ($\dot{V}E/\dot{V}O_{2}$) without an increase in $\dot{V}E$ relative to $\dot{V}{\text{CO}}_{2}$ ($\dot{V}E/\dot{V}O_{2}$); and (3) an increase in end tidal O_2_ tension without decreasing end tidal CO_2_ tension. The mean response time (MRT) of $\dot{V}O_{2}$ during ramp exercise was defined as the time between the beginning of the ramp test and the intersection between baseline $\dot{V}O_{2}$ ($\dot{V}O_{2b}$) and backwards extrapolation of the regression line of the $\dot{V}O_{2}$ – time relationship as described previously (Boone et al. 2008; Goulding et al. 2017). Work rates for subsequent tests were therefore calculated using the linear regression of the $\dot{V}O_{2}$– time relationship and solving for $\dot{V}O_{2}$, with account taken of the MRT.

### Experimental trials

For the following eight visits, participants were required to exercise to exhaustion at four fixed severe‐intensity power outputs each performed twice, once from a baseline of moderate intensity cycling, and once from a baseline of unloaded cycling. The power outputs of these severe‐intensity bouts of exercise were selected based upon performance during the incremental ramp test and were calculated to be in the range of 50%∆ (i.e., a work rate calculated to require 50% of the difference between the GET and $\dot{V}O_{2}\text{max}$ ) to 110% $\dot{V}O_{2}\text{max}$. The goal of this range of power outputs was to ensure that the tolerable duration of exercise was between 2 and 15 min in all cases, with at least 5 min separating the tolerable durations of the longest and shortest tests (Hill 1993). Furthermore, exercise durations <2 min were avoided because these have been shown to overestimate critical power (Mattioni Maturana et al. 2018). These power outputs are subsequently referred to as WR 1, WR 2, WR 3, and WR 4, with WR 1 being the lowest and WR 4 being the highest power outputs, respectively. On occasion, adjustments were made to the required power output of the subsequent exercise tests based upon performance in the initial tests. Participants alternated between conditions and the power outputs were presented in random order to prevent an order effect. Participants were not informed of their work rate or performance until the entire project had been completed. Each power output was performed once in each condition; that is, once with exercise initiated from an unloaded baseline (U→S), and once with exercise initiated from a moderate intensity baseline at 90% of the GET (M→S). U→S consisted of 3 min of unloaded baseline pedaling at 7 W (unloaded cycling on this ergometer elicits a power output of 7 W), after which an instantaneous step increase to the required severe‐intensity power output was abruptly applied, and participants exercised until the limit of tolerance. In M→S, participants performed 3 min of unloaded baseline pedaling at 0 W before an instantaneous step increase to a power output of 90% GET for 6 min (U→M). Subsequent to these 6 min of moderate‐intensity cycling, a further step increase in power output to the required severe intensity was abruptly applied, with participants exercising until they reached the limit of tolerance. $\dot{V}O_{2}$ peak for the constant work rate tests was defined as the mean $\dot{V}O_{2}$ value during the final 30 sec of exercise.

During all exercise tests, pulmonary gas exchange and ventilation were measured at the mouth breath‐by‐breath using a metabolic cart (Blue Cherry, Geratherm Respiratory, GmbH, Germany). Participants wore a silicone face mask (Hans Rudolph, Kansas) of known dead space attached to a low‐dead space flow sensor (Geratherm Respiratory, GmbH, Germany).The metabolic cart was connected to the participant via a capillary line connected to the flow sensor. The gas analyzers were calibrated before each test using gases of known concentrations and the flow sensors were calibrated using a 3‐L syringe (Hans Rudolph, Kansas City, MO). Heart rate was determined for every 1 sec during all tests using short‐range radiotelemetry (Garmin FR70, Garmin Ltd., Switzerland). For both U→S and M→S, blood was sampled from the thumb of the right hand into glass capillary tubes at rest, during the last minute of baseline pedaling preceding the severe‐intensity constant work rate bout, and immediately following exhaustion. Whole blood [L^−^] was determined using a Biosen lactate analyzer (Biosen C‐Line, EKF, Germany).

During experimental visits, continuous non‐invasive measurements of muscle oxygenation/deoxygenation status were made via a frequency‐domain multidistance near‐infrared spectroscopy (NIRS) system (Oxiplex TS, ISS, Champaign). The OxiplexTS uses one light‐emitting diode (LED) detector fiber bundle and eight LEDs functioning at wavelengths of 690 and 830 nm (four LEDs per wavelength). Light‐source detector separation distances of 2.25–3.75 cm for each wavelength were utilized with cell water concentration assumed constant at 70% and data sampled at 2 Hz. This NIRS device measures and incorporates the dynamic reduced scattering coefficients to provide absolute concentrations (*μ*mol/L) of deoxygenated hemoglobin + myoglobin ([HHb + Mb]), which is relatively unaffected by changes in blood volume during exercise (De Blasi et al. 1993; Ferrari et al. 1997; Grassi et al. 2003). NIRS has been demonstrated to produce valid estimates of O_2_ extraction (De Blasi et al. 1993; Ferrari et al. 1997; DeLorey et al. 2003; Grassi et al. 2003; Ferreira et al. 2006). However, the absorption spectrum of [Mb] converges with that of [HHb]; therefore at present, NIRS is unable to differentiate between the relative contributions of [Mb] and [HHb] to the overall NIRS signal (De Blasi et al. 1991). In referring to the NIRS deoxygenation signal as [HHb + Mb], the contribution of [Mb] is therefore also acknowledged. The NIRS device also provides measures of [oxygenated hemoglobin + myoglobin] ([HbO_2_ + MbO_2_]) and [total hemoglobin + myoglobin] ([THb + Mb]) concentration (as [HbO_2 _+ MbO_2_] + [HHb + Mb]) and thus, an indication of O_2_ availability. The flexible NIRS probe was placed longitudinally along the belly of the right vastus lateralis muscle midway between the greater trochanter and the lateral condyle of the tibia. The area underneath the probe was shaved and marked with washable pen such that the probe position could be replicated for each subsequent visit. The probe was held firmly in place by elastic Velcro strapping. Following each trial, depressions of the probe on the participant's skin were examined to confirm that the probe did not move during the trial, which was the case for every exercise transition. The NIRS probe was calibrated prior to each testing session using a calibration block of known absorption and scattering coefficients. Calibration was then verified using a second block of known but distinctly different absorption and scattering coefficients. Each of these procedures was according to the manufacturer's recommendations.

### Data analysis

The raw breath‐by‐breath $\dot{V}O_{2}$ data from each constant‐power exercise bout were first inspected to identify data points deemed atypical of the underlying response (i.e., due to coughs, swallows, sighs etc.) by removing data points lying more than four standard deviations from the local mean determined using a 5‐breath moving average. Edited $\dot{V}O_{2}$ data were then subsequently linearly interpolated to yield second‐by‐second values. For $\dot{V}O_{2}$ responses to the U→M transitions, the four identical transitions were ensemble‐averaged to produce a single dataset; the criterion severe‐intensity bouts for determination of critical power and W' in each condition were not repeated, therefore each were modeled separately. For each exercise transition, all data points preceding a sharp drop in respiratory exchange ratio and end‐tidal O_2_ pressure were omitted to exclude the phase I (cardiodynamic) component (Whipp and Ward 1990), and a single exponential equation (eq. (1)) with time delay was then fitted to the data:

(1) $$\dot{V}O_{2(t)}=\dot{V}O_{2(b)}+A_{VO_{2}}*\left(1-e^{-(t-TD_{VO_{2}}/\tau_{VO_{2}})}\right)$$

where $\dot{V}O_{2(t)}$ is the $\dot{V}O_{2}$ at any time *t*; $\dot{V}O_{2(b)}$ is the baseline $\dot{V}O_{2}$ which was taken as the mean $\dot{V}O_{2}$ from the last 30 sec of the baseline cycling period preceding the exercise bout, $A_{{\dot{V}}_{O_{2}}}$ is the amplitude of the fundamental response, $TD_{{\dot{V}}_{O_{2}}}$ is the time delay of the fundamental response relative to exercise onset, and $\tau_{\dot{V}O_{2}}$ is the time constant of the fundamental response. For U→M and U→S transitions, $\dot{V}O_{2(b)}$ was taken as the average $\dot{V}O_{2}$ from the final 30 sec of unloaded baseline cycling whereas for M→S, $\dot{V}O_{2(b)}$ was taken as the average $\dot{V}O_{2}$ from the final 30 sec of moderate intensity baseline cycling. For the U→M transitions, the exponential model was fitted to 360 sec. For U→S and M→S transitions, the onset of the $\dot{V}O_{2}$ slow component () was identified using purpose‐designed programming in Microsoft Excel (Microsoft Corporation, Redmond, WA) which iteratively fitted a single exponential function to the $\dot{V}O_{2}$ data, starting at 60 sec and extending the fitting window until the window encompasses the entire exercise bout. The resulting $\tau_{\dot{V}O_{2}}$ values were plotted against time, with the identified using the following criteria: (1) the point at which $\tau_{\dot{V}O_{2}}$ demonstrates a sustained increase from a previously “flat” profile, and (2) the demonstration of a local threshold in the *X* ^*2*^ value (Rossiter et al. 2001). This method allows the fitting of Equation (1) to the isolated fundamental component of the response before the slow component becomes discernible, thus avoiding the possibility of arbitrarily parameterizing the slow component. The isolated fundamental responses were then fitted with the same exponential function as shown in Equation (1) using Origin 6.0 (OriginLab Corporation, MA) to obtain the 95% confidence intervals for the derived parameter estimates. The $\dot{V}O_{2}$ slow component amplitude ($SC_{{\dot{V}}_{O_{2}}}$) was determined by calculating the difference between the end exercise $\dot{V}O_{2}$ (i.e., mean $\dot{V}O_{2}$ over final 30 sec of exercise) and ($A_{{\dot{V}}_{O_{2}}}$+ $\dot{V}O_{2(b)}$). In instances where exercise duration was too short to allow the slow component to be discerned (typically at WR 3 and 4 in M→S), the $\dot{V}O_{2}$ response was modeled using Equation (1) to the end of exercise and the slow component was assigned a value of 0. The functional gain of the fundamental $\dot{V}O_{2}$ response was also calculated by dividing $A_{{\dot{V}}_{O_{2}}}$ by the change in work rate (i.e., $A_{{\dot{V}}_{O_{2}}}$/Δ work rate).

The NIRS derived [HHb + Mb] responses to U→S and M→S exercise transitions were also modeled to provide information on the kinetics of muscle deoxygenation. Since [HHb + Mb] increases after a short delay following the onset of square‐wave exercise, the time delay before the onset of the exponential increase in [HHb + Mb] (i.e., TD_[HHb + Mb]_) was defined as when [HHb + Mb] had risen more than 1 SD above the mean baseline value obtained during the final 30 sec of baseline cycling (DeLorey et al. 2003). On occasions where [HHb + Mb] decreased after the exercise onset, TD_[HHb + Mb]_ was taken as the first point following the nadir showing a sustained increase in [HHb + Mb]. Data prior to this point were removed, and the [HHb + Mb] data was then fit with a single exponential equation (eq. (1)) of the form:

(2) $$\begin{array}{c} {}[ \end{array}\text{HHb + Mb}]\left(t\right)=\left[\text{HHb + Mb}\right]\left(b\right)+A\left[\text{HHb + Mb}\right]*(1-e\left(t-\text{TD}\left[H\text{Hb + Mb]}\right)\tau \left[\text{HHb+Mb}\right]\right)$$

where [HHb + Mb]_*(b)*_ is the mean [HHb + Mb] measured over the final 30 sec of baseline cycling, *A*_[HHb + Mb]_ is the asymptotic amplitude of the response, TD_[HHb + Mb]_ is the time delay relative to the onset of exercise and *τ*_[HHb + Mb]_ is the time constant for the response. The model fitting window was constrained to ${\text{TD}}_{S\dot{C}V_{O_{2}}}$. The amplitude of the [HHb + Mb] “slow component” was calculated by subtracting [HHb + Mb]_*(b) *_+ *A*_[HHb + Mb]_ from the mean value of [HHb + Mb] during the final 30 sec of exercise. To indicate changes in muscle oxygenation and total blood volume, the mean values for [HbO_2 _+ MbO_2_] and [THb + Mb] were determined at baseline (30 sec preceding each transition), at 30 sec and 120 sec into the exercise transition (15 sec bins centerd on 30 and 120 sec), and at end exercise (final 30 sec) to allow comparisons between conditions. These specific time points were selected to permit comparisons between conditions early in the transition (during the fundamental rise of $\dot{V}O_{2}$ before the onset of the $\dot{V}O_{2}$ slow component) and after the $\dot{V}O_{2}$ slow component had developed fully (i.e., at exercise termination). In addition to modeling the $\dot{V}O_{2}$ and [HHb + Mb] responses to exercise, we also calculated the ratio of change in [HHb + Mb] to change in $\dot{V}O_{2}$ (Δ[HHb + Mb]/Δ$\dot{V}O_{2}$) to provide further information on the degree of reliance on O_2_ extraction to satisfy a given $\dot{V}O_{2}$ in each transition. First, the second‐by‐second $\dot{V}O_{2}$ and [HHb + Mb] were linearly interpolated to give 5 sec averages, and these 5 sec averaged data were normalized for each transition (with 0% reflecting the baseline value and 100% reflecting the final amplitude of the fundamental response). The normalized $\dot{V}O_{2}$ data were then left‐shifted by the duration of phase I (previously determined) so that the fundamental phase $\dot{V}O_{2}$ increase was aligned with exercise onset. A mean value of the Δ[HHb + Mb]/Δ$\dot{V}O_{2}$ ratio was calculated for each transition as the average ratio value across the duration of the fundamental phase (Murias et al. 2011).

Heart‐rate kinetic responses to U→S and M→S exercise transitions were also modeled using a single exponential function (Eq. (2)) with the response constrained to the start of exercise (at *t *=* *0; i.e., with no time delay):

(3) HR(t)=HR(b)+AHR∗(1−e(t/τHR)

where HR_*(b)*_ is the mean HR measured over the final 30 sec of baseline cycling, *A*_HR_ is the asymptotic amplitude of the response and *τ*_HR_ is the time constant of the response. The fitting window was constrained to ${\text{TD}}_{S\dot{C}V_{O_{2}}}$.

Critical power and W′ were estimated using three models: the hyperbolic power‐time (P‐T) model (Eq. (3)); the linear work‐time (W‐T) model, where the total work done is plotted against time (Eq. (4)); and the linear inverse‐of‐time (1/T) model (eq. (5)), where power output is plotted against the inverse of time:

(4) $$P=W^{\prime }/T+\text{CP}$$

(5) $$W=\text{CP}*\text{T +}\;W^{\prime }$$

(6) $$P=W^{\prime }*\left(1/T\right)+\text{CP}$$

The standard errors of the estimates (SEE) associated with CP and W′ were expressed as a coefficient of variation (CV) relative to the parameter estimate. The total error associated with the obtained parameters was calculated as the sum of the CV associated with the critical power and W′ across both conditions for each individual participant. The sum of the CV was optimized for each individual by selecting the model with the lowest total error to produce the “best individual fit” parameter estimates. The model producing the best individual fit estimates was the same in both conditions for each participant.

### Statistical analyses

Two‐way repeated measures ANOVAs (condition * work rate) were used to compare differences in all kinetic parameters (i.e., $\dot{V}O_{2}$_,_ [HHb + Mb] and heart rate) and Δ[HHb + Mb]/Δ$\dot{V}O_{2}$. Three‐way repeated measures ANOVAs (condition * work rate * time) were used to compare differences in blood [L^−^], [HbO_2 _+ MbO_2_], and [THb + Mb] between tests. Planned repeated and simple contrasts were used to locate any significant main or interaction effects. Mauchly's test was used to test for the assumption of sphericity for repeated measures factors. Where this assumption was violated, the Greenhouse‐Geisser correction factor was applied to adjust the degrees of freedom. Student's paired *t* tests were used to compare differences in critical power and W' between conditions. Pearson's product‐moment correlation coefficient was used to assess the relationship between critical power and $\tau_{\dot{V}O_{2}}$. All data are presented as mean ± SD unless otherwise stated, and 95% confidence intervals (95% CI) are presented for modeled time constant parameters. For clarity, and to highlight values for parameters measured across all four severe‐intensity work rates, the overall mean across work rates ± SD is presented in text, with work rate‐specific mean ± SD presented in tables. Statistical significance was accepted at *P* < 0.05.

## Results

The mean $\dot{V}O_{2}\text{max}$ and peak work rate achieved during the incremental ramp test was 3.40 ± 0.66 L min^−1^ (43.0 ± 6.5 mL kg^−1^ min^−1^) and 312 ± 59 W, respectively. The GET occurred at 1.87 ± 0.30 L min^−1^ (118 ± 29 W), hence the U→M exercise transitions (90% GET) that formed part of the work‐to‐work condition were undertaken at 106 ± 26 W. There was no significant main effect of condition (*P *=* *0.325) or work rate (*P *=* *0.219) on blood [L^−^], however there was a significant main effect of time (*P *<* *0.001). Planned repeated contrasts revealed that blood [L^−^] was not different between rest and baseline (U→S rest = 1.46 ± 0.47 mmol L^−1^ vs. U→S baseline = 1.37 ± 0.46 mmol L^−1^; M→S rest = 1.39 ± 0.43 mmol L^−1^ vs. M→S baseline = 1.61 ± 0.31 mmol L^−1^), but increased significantly at end‐exercise (U→S end‐exercise = 11.56 ± 1.10 mmol L^−1^; M→S end‐exercise = 11.36 ± 1.87 mmol L^−1^). The lack of a significant condition * time interaction effect (*P *=* *0.184) indicates that there was no difference in blood [L^−^] between conditions during baseline cycling, and therefore the desired moderate intensity domain was achieved at baseline during M→S, and that this condition did not affect blood [L^−^] accumulation during the criterion exercise bouts.

**Figure 1:**
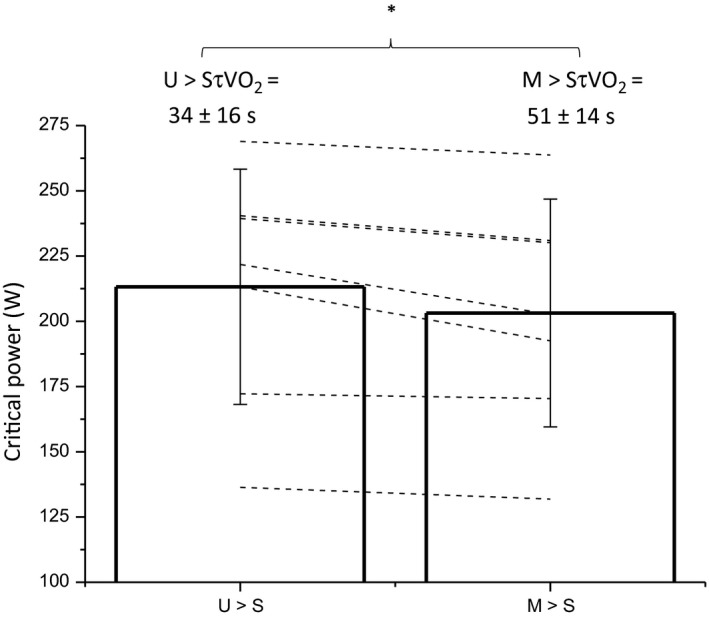
Critical power measured in the unloaded baseline (U > S) and elevated baseline (M > S) conditions. Group mean ± SD (*n *=* *7) are shown as open bars, and individual participant changes are shown as dashed black lines. * significant difference between conditions (*P *=* *0.011).

Individual fit optimization resulted in the hyperbolic power‐time model being used for 4 participants and the work‐time model being used for 3 participants. Critical power was reduced in M→S compared with U→S (U→S = 213 ± 45 W, CV = 3 ± 1% vs. M→S = 203 ± 44 W, CV = 3 ± 1%; *P *=* *0.011, Fig. 1), whereas there were no differences in W' between conditions (U→S = 15.6 ± 4.7 kJ, CV = 12 ± 5% vs. M→S = 16.1 ± 3.8 kJ, CV = 15 ± 8%; *P *=* *0.754). The $\tau_{\dot{V}O_{2}}$ determined from the U→M exercise transitions was 29 ± 13 sec. This was significantly inversely related to the critical power, normalized for body mass, as determined in the control condition (U→S) (*R* ^2^ = 0.90; *P *=* *0.001).

**Figure 2:**
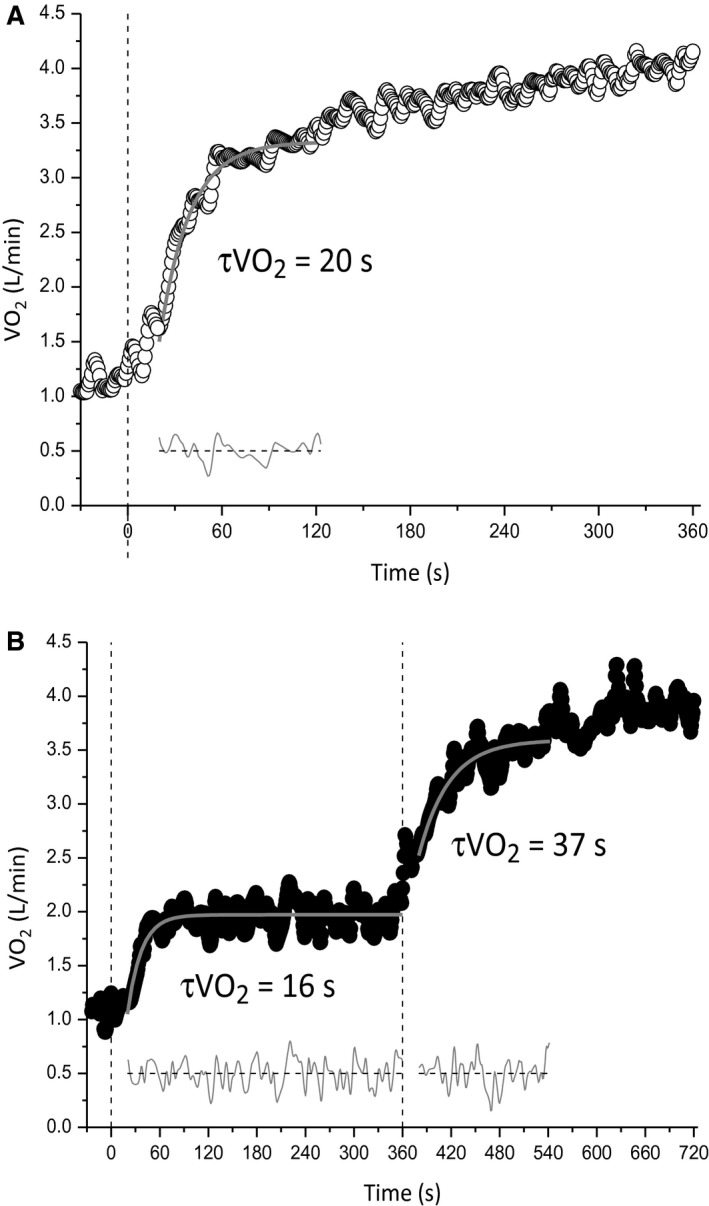
Pulmonary oxygen uptake ($\dot{V}O_{2}$) responses and best‐fit modelled responses of a representative participant in the unloaded baseline (A) and elevated baseline (B) conditions. $\tau_{\dot{V}O_{2}}$ values are displayed for each transition, with the thick gray lines representing the modeled fits. Lines of residuals are displayed at the bottom in gray. Vertical dashed black lines represent the onset of each step transition.

The parameters of the $\dot{V}O_{2}$ kinetics at each work rate for both conditions are presented in Table 1. The $\dot{V}O_{2}$ responses of a representative participant at a single work rate in both conditions and the respective modeled fits are shown in Figure 2. Baseline $\dot{V}O_{2}$ was significantly higher in M→S when compared with U→S (U→S = 0.94 ± 0.21 L min^−1^ vs. M→S = 1.72 ± 0.45 L min^−1^; *P *= 0.001) whereas $A_{{\dot{V}}_{O_{2}}}$(U→S = 1.94 ± 0.44 L min^−1^ vs. M→S = 1.37 ± 0.38 L min^−1^; *P *<* *0.001) and $SC_{{\dot{V}}_{O_{2}}}$ (U→S = 0.51 ± 0.25 L min^−1^ vs. M→S = 0.20 ± 0.15 L min^−1^; *P *=* *0.017) were reduced in M→S compared with U→S; end‐exercise $\dot{V}O_{2}$ did not differ between conditions or work rates (Table 1). $\tau_{\dot{V}O_{2}}$ was greater (U→S = 34 ± 15 sec, 95% CI 3 ± 2 sec, vs. M→S = 51 ± 15 sec, 95% CI 5 ± 3 sec; *P *=* *0.002) in M→S compared with U→S.

**Table 1:** Pulmonary oxygen uptake and muscle deoxyhemoglobin + myoglobin responses to severe‐intensity constant work rate exercise in each condition

	$\dot{V}O_{2}$		[HHb + Mb]	
Baseline (L min^−1^/*μ*M)	U→S	M→S	U→S	M→S
	*		*	
WR 1	0.97 ± 0.24	1.73 ± 0.43	15.34 ± 4.79	20.26 ± 8.32
WR 2	0.97 ± 0.18	1.77 ± 0.41	18.44 ± 5.63	19.60 ± 7.76
WR 3	0.87 ± 0.21	1.70 ± 0.54	16.89 ± 5.34	20.59 ± 8.62
WR 4	0.97 ± 0.20	1.72 ± 0.41	15.34 ± 4.50	17.71 ± 7.72
TD (sec)	*		*	
WR 1	12 ± 6	7 ± 8	10 ± 4	17 ± 14
WR 2	16 ± 7	10 ± 8	11 ± 4	17 ± 14
WR 3	15 ± 10	12 ± 10	10 ± 5	9 ± 3
WR 4	13 ± 4	8 ± 5	7 ± 3	8 ± 5
*τ* (sec)	*		*	
WR 1	38 ± 23	49 ± 17	20 ± 15	28 ± 17
WR 2	29 ± 13	55 ± 15	11 ± 4	31 ± 17
WR 3	35 ± 12	46 ± 17	16 ± 12	27 ± 13
WR 4	33 ± 11	52 ± 11	9 ± 2	22 ± 7
95% CI *τ* (sec)				
WR 1	3 ± 2	6 ± 3	5 ± 3	6 ± 4
WR 2	2 ± 1	6 ± 3	2 ± 2	5 ± 2
WR 3	3 ± 2	5 ± 3	3 ± 2	6 ± 3
WR 4	3 ± 1	6 ± 3	2 ± 1	5 ± 2
*A* (L min^−1^/μM)	*#		*	
WR 1	1.78 ± 0.34	1.07 ± 0.35	6.41 ± 5.99	4.51 ± 3.23
WR 2	1.80 ± 0.40	1.23 ± 0.31	7.09 ± 3.53	5.28 ± 3.43
WR 3	2.07 ± 0.45	1.50 ± 0.49	7.90 ± 6.38	6.26 ± 3.11
WR 4	2.09 ± 0.57	1.67 ± 0.37	9.31 ± 8.68	5.33 ± 3.29
Absolute *A* (L min^−1^/*μ*M)	*			
WR 1	2.75 ± 0.49	2.80 ± 0.69	21.75 ± 10.04	24.77 ± 11.33
WR 2	2.77 ± 0.50	3.00 ± 0.65	25.53 ± 8.97	24.88 ± 10.82
WR 3	2.95 ± 0.58	3.21 ± 0.75	26.44 ± 10.41	26.85 ± 11.51
WR 4	3.06 ± 0.71	3.39 ± 0.68	24.65 ± 11.94	23.04 ± 10.15
Gain, mL min^−1^ W^−1^/Δ[HHb + Mb]/Δ$\dot{V}O_{2}$				*
WR 1	8.06 ± 1.31	8.33 ± 1.97	1.10 ± 0.14	0.84 ± 0.22
WR 2	7.43 ± 0.42	8.11 ± 1.37	1.17 ± 0.11	0.91 ± 0.23
WR 3	7.66 ± 0.71	8.39 ± 1.44	1.19 ± 0.14	0.92 ± 0.22
WR 4	6.93 ± 0.90	8.27 ± 1.26	1.38 ± 0.21	1.04 ± 0.11
SC (L min^−1^/*μ*M)	*			
WR 1	0.56 ± 0.30	0.43 ± 0.17	6.12 ± 7.06	6.94 ± 5.33
WR 2	0.72 ± 0.16	0.34 ± 0.33	6.85 ± 4.76	4.63 ± 4.70
WR 3	0.54 ± 0.33	0.04 ± 0.11	2.05 ± 1.48	1.76 ± 2.10
WR 4	0.21 ± 0.21	0.00 ± 0.00	0.86 ± 1.22	2.14 ± 2.33
End‐ex (L min^−1^/*μ*M)				
WR 1	3.32 ± 0.68	3.24 ± 0.70	28.15 ± 15.48	31.52 ± 16.01
WR 2	3.49 ± 0.47	3.34 ± 0.43	31.30 ± 11.23	28.63 ± 13.49
WR 3	3.46 ± 0.77	3.23 ± 0.84	26.44 ± 10.58	28.61 ± 11.77
WR 4	3.24 ± 0.67	3.19 ± 0.68	24.37 ± 11.93	24.28 ± 9.80

$\dot{V}O_{2}$, oxygen uptake; [HHb + Mb], deoxyhemoglobin +  myoglobin; baseline, average value over final 30 sec of baseline period; TD, fundamental time delay; *τ*, fundamental time constant; *A*, fundamental amplitude; Absolute *A*, baseline + fundamental amplitude; Gain, increase in fundamental phase $\dot{V}O_{2}$ per unit increase in power output; Δ[HHb + Mb]/Δ$\dot{V}O_{2}$, ratio of change in [HHb + Mb] to change in $\dot{V}O_{2}$; SC, magnitude of the slow component; end‐ex, average value over final 30 sec of exercise. Absolute [HHb + Mb] data presented in *μ*mol/L, $\dot{V}O_{2}$ data presented in L min^−1^ * indicates significant main effect of condition, # indicates significant main effect of work rate (*P *<* *0.05).

**Figure 3:**
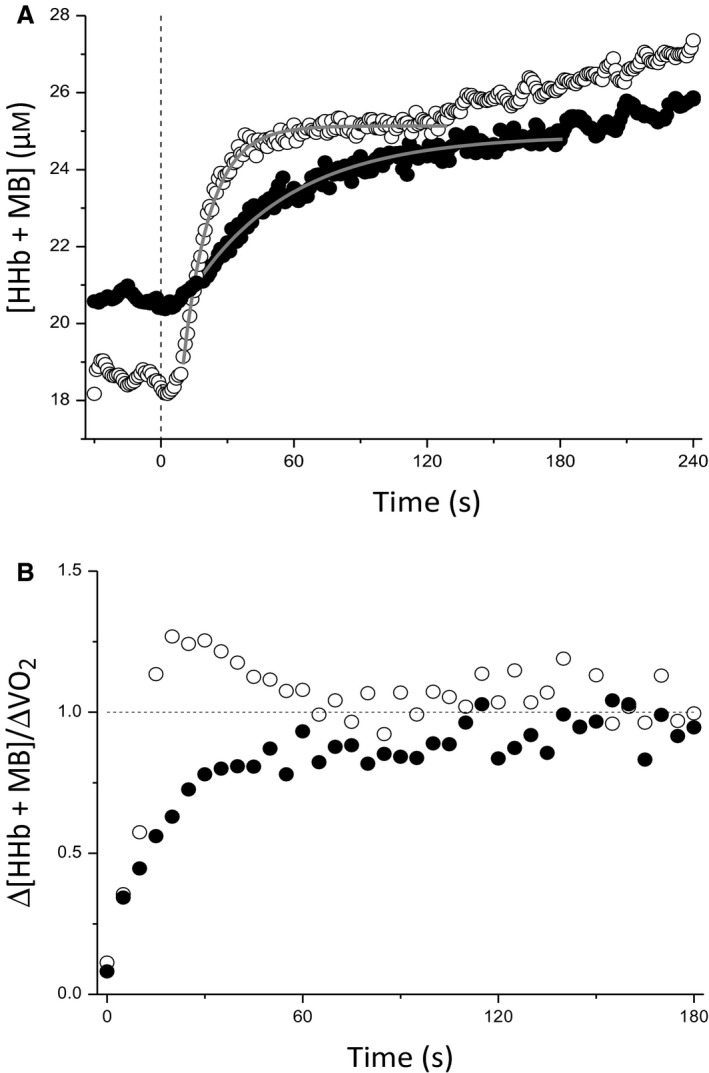
Group mean ± SD muscle deoxyhemoglobin + myoglobin ([HHb + Mb]) responses to severe exercise at work rate 2 in the U > S (clear circles) and M > S (black circles) conditions (*n *=* *7) (A). The group mean modeled fits are transposed as solid gray lines. The vertical dashed black line represents the onset of each step transition. Error bars omitted for clarity. Panel (B) demonstrates the transient profiles of Δ[HHb + Mb]/Δ$\dot{V}O_{2}$ in U > S (clear circles) and M > S (black circles). Horizontal dashed line represents the “steady state” value of 1.0.

**Figure 4:**
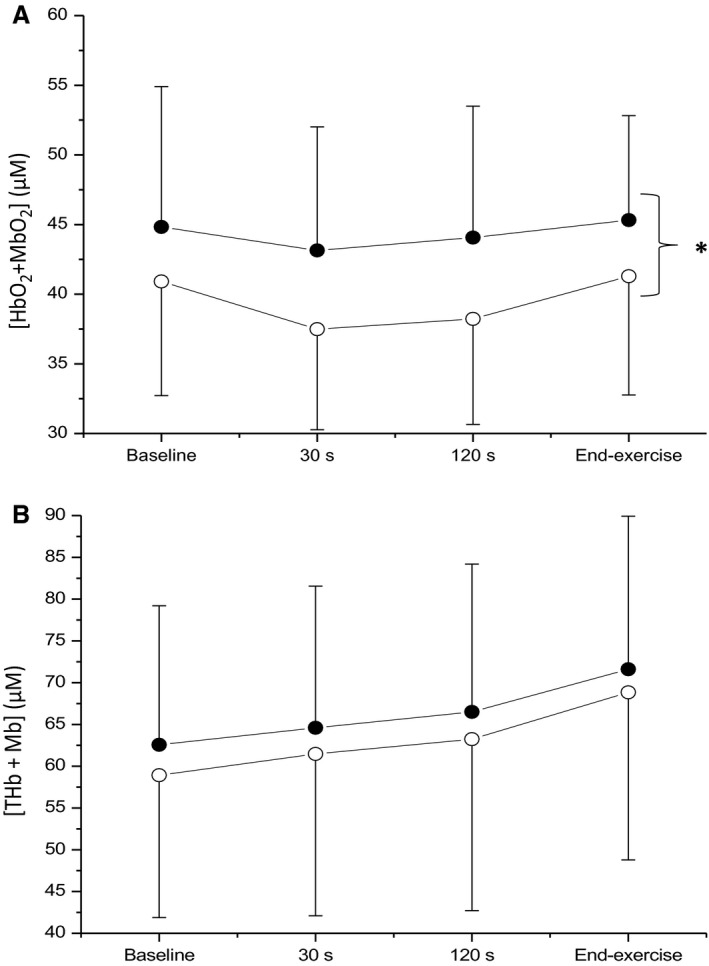
Comparisons of group mean oxyhemoglobin + oxymyoglobin ([HbO_2_ + MbO_2_]) (A) and total hemoglobin + myoglobin ([THb + Mb]) (B) across all work rates in each condition. Open circles represent unloaded baseline condition whereas black circles represent elevated baseline condition. * indicates significant main effect of condition (*P *=* *0.031).

The parameters of the [HHb + Mb] kinetics and Δ[HHb + Mb]/Δ$\dot{V}O_{2}$ analysis at each work rate for both conditions are presented in Table 1; the group mean [HHb + Mb] responses to exercise at a single work rate (WR 2) in each condition are depicted in Figure 3A; whereas the group mean transient profiles of Δ[HHb + Mb]/Δ$\dot{V}O_{2}$ at the same work rate are displayed in Figure 3B. Baseline [HHb + Mb] was greater in M→S compared with U→S (U→S = 16.1 ± 5.0 *μ*mol L^−1^ vs. M→S = 19.5 ± 7.7 *μ*mol L^−1^; *P *=* *0.007). *τ*_[HHb + Mb]_ was greater when determined in M→S in comparison to U→S (U→S = 14 ± 7 sec, 95% CI 3 ± 1 sec vs. M→S = 27 ± 7 sec, 95% CI 7 ± 2 sec; *P *=* *0.007), the Δ[HHb + Mb]/Δ$\dot{V}O_{2}$ ratio (U→S = 1.20 ± 0.11 vs. M→S = 0.96 ± 0.10; *P *<* *0.001) was smaller in M→S when compared with U→S, and end‐exercise [HHb + Mb] did not differ between conditions or work‐rates (Table 1). The group mean [HbO_2 _+ MbO_2_] and [THb + Mb] responses to exercise at a representative work rate are illustrated in Figure 4. [HbO_2 _+ MbO_2_] was significantly greater in M→S when compared with U→S (*P *=* *0.031, Fig. 4, panel A), however there was no difference between conditions in [THb + Mb] (*P *=* *0.114, Fig. 4, panel B). Baseline heart rate (U→S = 99 ± 17 beats min^−1^ vs. M→S = 120 ± 19 beats min^−1^; *P *=* *0.003) and *τ*_HR_ (U→S = 47 ± 22 sec, 95% CI 3 ± 2 sec, vs. M→S = 75 ± 30 sec, 95% CI 3 ± 2 sec; *P *=* *0.009) were both greater in M→S when compared with U→S.

## Discussion

The purpose of the present study was to determine whether $\tau_{\dot{V}O_{2}}$ is an independent determinant of critical power by manipulating the speed of the pulmonary $\dot{V}O_{2}$ kinetics without altering O_2_ availability, and subsequently observing the effect on critical power. We hypothesized that $\dot{V}O_{2}$ kinetics would be slower (i.e., increased $\tau_{\dot{V}O_{2}}$) and critical power lower when exercise was initiated from an elevated, compared to an unloaded, baseline work rate during upright cycle exercise. Our findings were consistent with both of these hypotheses. Crucially, NIRS‐derived measures of O_2_ availability did not suggest that the slowed $\dot{V}O_{2}$ kinetics were due to insufficient O_2_ availability when exercise was initiated from an elevated baseline work rate. These results therefore demonstrate for the first time that $\tau_{\dot{V}O_{2}}$, a measure of the rate of adaptation of $\dot{V}O_{2}$ at exercise onset, is an independent physiological determinant of critical power, which has previously been shown to represent the upper limit for the attainment of a metabolic steady state (Poole et al. 1988; Jones et al. 2008).

### Effects of oxygen uptake kinetics on critical power

In this study, we observed a ~50% increase of $\tau_{\dot{V}O_{2}}$ when exercise was initiated from an elevated baseline work rate (M→S) compared with a baseline of unloaded cycling (U→S), consistent with previous research (Hughson and Morrissey 1982; di Prampero et al. 1989; Brittain et al. 2001; Wilkerson and Jones 2007; DiMenna et al. 2008, 2010a; Bowen et al. 2011; Breese et al. 2013). This slowing of the $\dot{V}O_{2}$ kinetics occurred with a concomitant ~10 W reduction in critical power. Additionally, and also consistent with previous research (Murgatroyd et al. 2011; Goulding et al. 2017), we observed a strong inverse relationship (*R* ^2^ = 0.90) between $\tau_{\dot{V}O_{2}}$ and critical power. Since the NIRS data suggest that the increase in $\tau_{\dot{V}O_{2}}$ in M→S compared with U→S was not due to insufficient O_2_ availability (see below), collectively, the data suggest that $\tau_{\dot{V}O_{2}}$ is an independent determinant of critical power.

Because critical power represents the upper limit for which a metabolic steady state is obtainable (Poole et al. 1988; Jones et al. 2008; Vanhatalo et al. 2016), critical power may be regarded as the highest work rate for which the O_2_ deficit and mechanical efficiency can be stabilized. For a given power output, $\tau_{\dot{V}O_{2}}$ is the primary determinant of the size of the O_2_ deficit, with more rapid $\dot{V}O_{2}$ kinetics enabling a smaller O_2_ deficit to be incurred (Whipp et al. 1982). Consequently, the greater $\tau_{\dot{V}O_{2}}$ observed in M→S may have reduced critical power by virtue of a reduction in the highest power output for which the O_2_ deficit could be stabilized. Inherent within this interpretation, is that the magnitude of metabolic perturbation during the rest‐to‐exercise transition determines whether or not a steady state can be attained. Presumably beyond some “critical” level of metabolic perturbation there is a continued reduction in mechanical efficiency, thus preventing the attainment of a steady state. Hence the slower $\dot{V}O_{2}$ kinetics observed during M→S, would exacerbate the degree of metabolic perturbation during a given exercise transition and cause a reduction in the highest power output for which a steady state can be obtained.

The signaling processes that cause the inability to attain a metabolic steady state during exercise above the critical power likely relate to the accumulation of the products of ATP breakdown. [ADP] is below its Michaelis constant (*K*_m)_ in resting skeletal muscle, and its increase as a consequence of ATP breakdown causes large increases in the rate of oxidative phosphorylation (Chance and Williams 1955; Connett et al. 1990; Honig et al. 1992), with the relationship between [ADP] and $\dot{V}O_{2}$ previously observed as hyperbolic (Jeneson et al. 1996; Glancy et al. 2008; Schmitz et al. 2012) (or sigmoidal in vivo; Wüst et al. 2011). However, high‐intensity exercise also results in a decline in muscle efficiency due to an increase in ATP turnover per unit of work performed (Zoladz et al. 2008; Cannon et al. 2011; Vanhatalo et al. 2011) and/or additional recruitment of inefficient type II fibers (Krustrup et al. 2004a,b, 2008), each causing further rises in [ADP]. These combined effects of high‐intensity exercise result in [ADP] encroaching further toward the flat region of the [ADP]‐$\dot{V}O_{2}$ relationship, where $\dot{V}O_{2}$ becomes progressively less sensitive to further increases in [ADP]. Critical power, therefore, might represent the work rate at which some “critical” [ADP] is achieved during the rest‐exercise transition. Once this critical [ADP] has been attained, the loss of sensitivity of $\dot{V}O_{2}$ to rising [ADP] is such that there is a continually increasing reliance on non‐oxidative metabolism and thus the inability to attain a physiological steady state. In turn, this triggers a cascade of fatigue‐related physiological events that eventually result in task failure (Demarle et al. 2001), including increased muscle ATP demand (Rossiter et al. 2002), progressive recruitment of additional motor units (Shinohara and Moritani 1992; Barstow et al. 1996; Scheuermann et al. 2001; Krustrup et al. 2004a,b; Endo et al. 2007), and the attainment of $\dot{V}O_{2}\text{max}$ max (Poole et al. 1988).

In this study therefore, the greater $\tau_{\dot{V}O_{2}}$ observed in M→S compared with U→S would have led to a greater rise in [ADP] for any given increase in power output due to increased reliance on non‐oxidative phosphorylation. Hence, the concomitant reduction in critical power in M→S compared to U→S is a consequence of the greater $\tau_{\dot{V}O_{2}}$ reducing the power output at which some critical level of [ADP], and thus a physiological steady state, is attained. When considered in the context of the current hypothesis therefore, $\tau_{\dot{V}O_{2}}$ is an independent determinant of critical power, because a smaller $\tau_{\dot{V}O_{2}}$ would lessen the disturbances in intracellular [ADP] during the exercise transition, thus permitting a physiological steady state to be attained at a higher power output and raising critical power.

### Mechanistic bases of the work‐to‐work exercise effect

It has previously been suggested that the slower fundamental phase $\dot{V}O_{2}$ kinetics observed with work‐to‐work exercise are the result of a slowed rate of adaptation of O_2_ delivery (Hughson and Morrissey 1982, 1983). However, in the present study, and consistent with previous literature (Spencer et al. 2011; Breese et al. 2013; Williams et al. 2013; Keir et al. 2014, 2016; Wüst et al. 2014; Nederveen et al. 2017), we found that *τ*_[HHb + Mb]_ was greater in M→S when compared with U→S. The NIRS‐derived [HHb + Mb] signal represents the relative balance between O_2_ delivery and utilization in the microvasculature within the field of interrogation. Hence the greater *τ*_[HHb + Mb]_ reflects slower muscle deoxygenation kinetics and thus indicates a greater O_2_ availability relative to the increasing demand for $\dot{V}O_{2}$ during the exercise transient in M→S compared with U→S. Furthermore, we observed a lower Δ[HHb + Mb]/Δ$\dot{V}O_{2}$ ratio, and an elevated [HbO_2 _+ MbO_2_] in M→S compared with U→S during the severe exercise bout. Taken together therefore, our data derived from NIRS are suggestive of a relative improvement in O_2_ availability and thus better matching of microvascular O_2_ distribution to muscle O_2_ utilization in the active tissues during M→S compared with U→S.

Given that our data do not implicate an O_2_ delivery limitation as the mechanism responsible for the slower fundamental phase $\dot{V}O_{2}$ kinetics observed with work‐to‐work exercise, the physiological mechanisms contributing to the slowed $\dot{V}O_{2}$ dynamics in M→S likely reflect the direct influence of a raised metabolism and/or the recruitment of higher order muscle fibers at the onset of severe exercise (Brittain et al. 2001; DiMenna et al. 2010a). The elevation in metabolic rate prior to the severe‐intensity exercise transition in M→S will reduce the cellular energetic state in already active fibers (i.e., reduced *P*O_2_ and [PCr], increased [ADP] and [P_i_], and less negative changes in the Gibbs free energy of ATP hydrolysis [Δ*G*_ATP_]), thus slowing the $\dot{V}O_{2}$ response in the subsequent exercise transition (Meyer and Foley 1996). Alternatively, Henneman's size principle (Henneman and Mendell 1981) predicts that motor units are recruited in an orderly fashion, with smaller, more oxidative fibers recruited first. Thus, when an exercise transition is made from an elevated baseline work rate, the newly recruited muscle fibers will reside at the higher end of the recruitment hierarchy, particularly during transition to severe‐intensity exercise. These muscle fibers have a greater O_2_ cost of contraction and possess slower $\dot{V}O_{2}$ kinetics relative to the smaller, more oxidative fibers (Crow and Kushmerick 1982; Willis and Jackman 1994; Reggiani et al. 1997; Behnke et al. 2003), and thus could also account for the slowing of the $\dot{V}O_{2}$ response in M→S.

Whether muscle fiber recruitment patterns and/or raised muscle metabolism were primarily responsible for the slowed $\dot{V}O_{2}$ kinetics during the work‐to‐work exercise is presently unclear, and may be intensity‐domain dependent (DiMenna et al. 2010a; Bowen et al. 2011);. Furthermore, the present study does not permit conclusions regarding the mechanisms underpinning the slower $\dot{V}O_{2}$ kinetics during M→S compared with U→S. Hence, both potential mechanisms may independently account for the greater $\tau_{\dot{V}O_{2}}$, and thus critical power, in M→S. However, regardless of which of these mechanisms was responsible for the slower $\dot{V}O_{2}$ kinetics in M→S, the present data suggest that the slowing of $\dot{V}O_{2}$ kinetics observed when exercise was initiated from an elevated baseline reduced critical power independently of O_2_ availability, demonstrating that $\tau_{\dot{V}O_{2}}$ is an independent determinant of critical power.

### Limitations

The use of single transitions to characterize the $\dot{V}O_{2}$ responses to exercise presents a limitation for the present study, as we were unable to improve the signal‐to‐noise ratio through the use of repeat transitions. However, repeat transitions were not feasible due to the undue requirements this would place on participants; repeating each transition would also create a greater risk of a training effect confounding the results. Despite this, the 95% CI associated with the $\tau_{\dot{V}O_{2}}$ was ~4 sec; a level of confidence that was likely aided by the high amplitude of the individual $\dot{V}O_{2}$ responses, which would have increased the signal‐to‐noise ratio in each transition. Additionally, the level of confidence reported in the $\tau_{\dot{V}O_{2}}$ parameter in the present study is less than the recently suggested minimally important difference to determine significant changes in intervention studies (Benson et al. 2017), and the 95% CI's were smaller than the mean difference in $\tau_{\dot{V}O_{2}}$ at all four work‐rates. Therefore, despite the use of single transitions at each work rate, we are confident in our reported effects of baseline work rate on $\tau_{\dot{V}O_{2}}$. Finally, our NIRS measurements were taken from a single site on a superficial portion of the vastus lateralis muscle. Muscle perfusion and deoxygenation are spatially heterogeneous during cycle exercise (Koga et al. 2007), therefore it is possible that O_2_ availability was limiting in other muscle regions that were not interrogated. However, type II fibers are more prevalent in superficial muscle (Johnson et al. 1973), and these muscle fibers rely more on elevated diffusive as opposed to perfusive O_2_ transport strategies to satisfy an increase in metabolic demand when compared with deeper muscle (Koga et al. 2017). Therefore, if any O_2_ delivery limitations were present, we would have been more likely to have detected them in the superficial region of the vastus lateralis that we interrogated, as opposed to deeper muscle groups.

## Conclusions

In conclusion, this study provides the first direct experimental evidence that $\tau_{\dot{V}O_{2}}$ is an independent determinant of critical power. Specifically, when exercise was initiated from an elevated baseline work rate, $\dot{V}O_{2}$ kinetics were slower and critical power was reduced compared to when exercise was initiated from a baseline of unloaded cycling. Crucially, the data derived from NIRS during exercise suggest that the slowing of the $\dot{V}O_{2}$ kinetics and concomitant decrease in critical power observed in M→S compared to U→S were not the result of an O_2_ availability limitation. Hence, our data demonstrate the independence of $\tau_{\dot{V}O_{2}}$ as a determinant of the upper work‐rate limit for the attainment of a physiological steady state, otherwise known as critical power, providing empirical support for the notion that “… *the seeds of exercise intolerance are sown from the very outset of exercise*” (Rossiter 2011).

## Conflicts of Interest

The authors declare no conflicts of interest.
